# Intramammary 25-hydroxyvitamin D_3_ and 1,25-dihydroxyvitamin D_3_ treatments differentially increase serum calcium and milk cell gene expression

**DOI:** 10.3168/jdsc.2022-0336

**Published:** 2022-12-14

**Authors:** T.L. Wells, M.B. Poindexter, M.F. Kweh, L.P. Blakely, C.D. Nelson

**Affiliations:** Department of Animal Sciences, University of Florida, Gainesville 32611

## Abstract

Intramammary 25-hydroxyvitamin D_3_ (25D) and 1,25-dihydroxyvitamin D_3_ (1,25D) treatments stimulate immune defenses of the mammary gland. We hypothesized 25D treatment, in contrast to 1,25D, would exert activity in the mammary gland without affecting serum calcium. The objective was to determine the effect of dose and source of intramammary vitamin D treatments on milk somatic cell gene expression and serum calcium. Twenty lactating Holstein cows with somatic cell count <200,000 cells/mL of milk were used for the experiment. Cows were blocked by somatic cell count and randomly assigned to 1 of 5 intramammary treatments (n = 4 cows/treatment): placebo control (CNTRL; 0.4% Tween 20 in phosphate-buffered saline), 100 μg of 25D, 500 μg of 25D, 10 μg of 1,25D, or 50 μg of 1,25D. Treatments were administered in 2 ipsilateral quarters after milking. Blood samples were collected at 0, 12, 24, and 48 h for measurement of Ca and 1,25D. Milk samples were collected from each quarter at 0, 6, 12, 24, and 48 h relative to the start of treatments for measurement of gene expression in milk somatic cells. The 1,25D treatments increased serum concentrations of 1,25D and Ca in a dose-dependent manner with maximum 1,25D and Ca concentrations of 199 ± 6 pg/mL and 2.73 ± 0.04 m*M*, respectively, observed for 50 μg of 1,25D cows compared with 59 ± 6 pg/mL and 2.54 m*M*, respectively, for CNTRL cows. The 25D treatments did not affect serum 1,25D and Ca compared with CNTRL. The 25D and 1,25D treatments increased mRNA transcripts for vitamin D 24-hydroxylase (*CYP24A1*), inducible nitric oxide synthase (*NOS2A*), and chemokine C-C motif ligand 5 (*CCL5*) in a dose-dependent manner. The 50 μg of 1,25D treatment resulted in the greatest *CYP24A1* expression (303-fold relative to CNTRL) at 6 h but was not different from CNTRL at 24 h. In contrast, *CYP24A1* was 57-fold greater for cows that received 500 μg of 25D compared with CNTRL at 24 h. In conclusion, intramammary 25D treatment is effective at regulating gene expression in the mammary gland without systemic effects on serum 1,25D and Ca that occur with intramammary 1,25D treatment.

## Highlights

• Intramammary 1,25-dihydroxyvitamin D increased serum Ca concentrations compared with serum 25-hydroxyvitamin D and placebo.
• Intramammary 25-hydroxyvitamin D did not increase serum Ca concentration compared with placebo.
• Intramammary 25-hydroxyvitamin D and 1,25-dihydroxyvitamin D treatments increased milk somatic cell *CYP24A1* and *NOS2A* expression.

## Graphical Abstract

**Figure:**
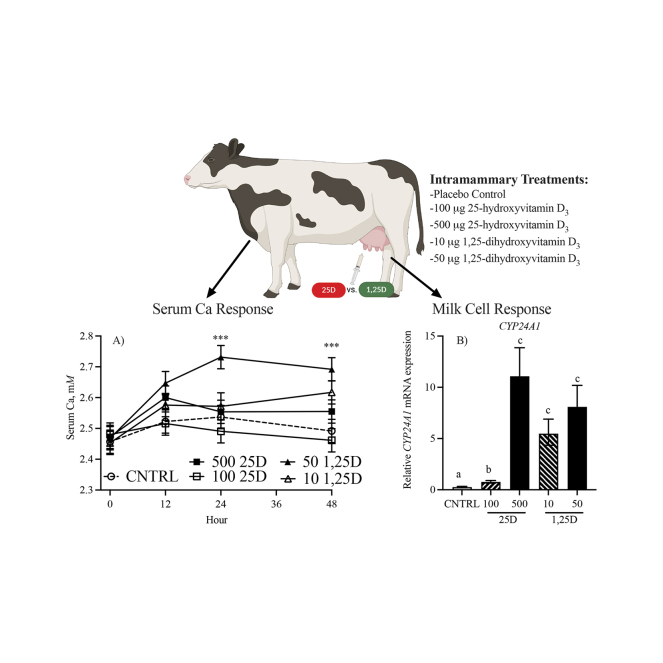
**Summary:** The objective was to determine the effect of dose and source of intramammary vitamin D treatments on milk somatic cell gene expression and serum calcium. Twenty lactating Holstein cows were assigned to 1 of 5 intramammary treatments (n = 4 cows/treatment): placebo control (10 mL of 0.4% Tween 20 in PBS), 100 μg of 25-hydroxyvitamin D3 (25D), 500 μg of 25D, 10 μg of 1,25-dihydroxyvitamin D3 (1,25D), or 50 μg of 1,25D. Intramammary 1,25D, but not 25D, increased serum Ca concentrations compared with placebo. Intramammary 25-hydroxyvitamin D and 1,25-dihydroxyvitamin D treatments increased milk somatic cell *CYP24A1* and *NOS2A* expression. Intramammary 25D exhibited biological activity in mammary gland without affecting serum Ca.

Mastitis in dairy cows remains as a significant threat to sustainable dairy production despite adoption of effective mastitis prevention plans (Ruegg, 2012). The bovine mammary gland is equipped with robust immune defenses such as phagocytes and antimicrobial proteins that protect against IMI that cause mastitis (Sordillo, 2018). However, elusive pathogens or breakdown in defense mechanisms leading to mastitis still occurs at a problematic rate. A better understanding of antibacterial defenses of the mammary gland and alternative therapies are needed to reduce the use of antibiotics in dairy cattle.

Intramammary vitamin D therapy offers a potential alternative or adjunctive therapy to current antimicrobial treatments for mastitis in cows (Lippolis et al., 2011). Activation of vitamin D signaling in immune cells is part of the innate immune response (Adams and Hewison, 2008) and dietary vitamin D has been shown to lessen the effects of mastitis (Poindexter et al., 2020) and incidence of metritis (Martinez et al., 2018). Vitamin D treatments upregulated inducible nitric oxide synthase (*NOS2A*) and β-defensin (i.e., *DEFB7*) in bovine monocytes (Merriman et al., 2015) and reduced *Staphylococcus aureus* infection of bovine mammary epithelial cells (Yue et al., 2017). Intramammary injection of 10 μg of 1,25D increased expression of *NOS2A*, *DEFB7*, and the gene encoding 25-hydroxyvitamin D 24-hydroxylase (*CYP24A1*) in milk somatic cells of healthy and subclinically infected mammary quarters (Merriman et al., 2017). Moreover, 100 μg of intramammary 25D reduced the severity of experimental *Streptococcus uberis* mastitis in cows (Lippolis et al., 2011).

In theory, immune cell 1α-hydroxylases will convert 25D to 1,25D without exerting systemic effects of 1,25D, namely increased serum Ca (Hewison, 2010); thereby making intramammary 25D treatment a safer but equally effective therapy compared with 1,25D. We hypothesized that 25D treatment, in contrast to 1,25D, would alter gene expression in the mammary gland without affecting serum calcium. The objective was to determine the effect of dose and source (25D vs. 1,25D) of intramammary vitamin D treatment on milk somatic cell gene expression and serum calcium.

The experimental procedures and care and treatment of animals were approved by the University of Florida Institutional Animal Care and Use Committee protocol # 201408677. The cows were enrolled in the experiment between May and June 2017. A sample size calculation indicated that a difference in serum Ca of 0.15 m*M* with a standard deviation of 0.05 m*M* could be detected with 4 cows per treatment in a 2-way ANOVA with α = 0.05 and β = 0.80. The experiment used 20 lactating (386 ± 90 DIM) and pregnant Holstein cows with parity <3, no cases of mastitis in last 30 d, and SCC <200,000 cell/mL. Average milk yield was 23 ± 8 kg/d and SCC was 91 ± 45 cells/μL. Cows were housed together in a sand-bedded freestall barn equipped with fans and sprinklers and fed a corn-silage based TMR providing 0.5 mg of cholecalciferol per 20 kg of DM as previously described (Poindexter et al., 2020).

The experiment was a randomized complete block design with 5 treatments in 4 blocks. Cows were blocked by SCC then randomly assigned the following treatments: placebo control (**CNTRL**; 0.4% Tween 20 in PBS), 100 μg of 25D, 500 μg of 25D, 10 μg of 1,25D, or 50 μg of 1,25D. Randomization was accomplished with the random function in Excel (Microsoft Corp.). The 25D and 1,25D solutions were prepared from purified powder concentrates (Cayman Chemical) and dissolving in reagent grade ethanol (Fisher Scientific) to a stock concentration of 1 mg/mL. The purity of the 25D and 1,25D concentrates was verified by detection of UV absorption from 200 to 300 nm using a spectrophotometer (Biotek Synergy HT). Purity was determined using a 228 to 264 nm ratio ≥1.5. The placebo, 25D, and 1,25D solutions were aseptically administered via a teat cannula into 2 ipsilateral mammary glands so that each cow received duplicate treatments. For cows receiving 25D or 1,25D treatments, the 2 quarters opposite treated quarters also received placebo injections so that responses to treatment within cow could also be measured. Treatments were administered at the completion of the morning milking. Researcher personnel were not blinded to treatments.

Serum samples were collected from the coccygeal blood vessels using evacuated 10-mL serum separator tubes (Vacutainer, Becton Dickinson) at 0.25, 12, 24, and 48 h relative to the start of treatments. The tubes were centrifuged at 1,500 × *g* for 20 min at 4°C for serum separation. The serum samples were transferred into microtubes and stored frozen at −20°C. Serum Ca concentrations were measured using an automated chemical analyzer (RX Daytona, Randox Laboratory Ltd.) according to the manufacturer's instructions. Concentrations of 1,25D in serum were analyzed by ELISA [1α,25(OH)_2_ Vitamin D ELISA; IBL-America] according to the manufacturer's instructions.

Milk was sampled from each quarter at 0, 6, 12, 24, and 48 h relative to the start of treatments for analysis of SCC and gene expression in milk somatic cells. Teats were stripped briefly to remove foremilk before collection. Samples were collected using 50-mL conical tubes (Fisher Scientific) before milk withdrawal from the milk machine and transported to the laboratory on ice. Complete removal of milk by machine was performed after sample collections at 0, 12, 24, and 48 h relative to treatments. Somatic cell counts and the proportion of cells that were neutrophils or macrophages in a 200-μL sample of milk were measured by flow cytometry using phycoerythrin-Cy5.5-conjugated anti-CD14 (Tük4 clone, Thermo Fisher Scientific) and fluorescein isothiocyanate-conjugated anti-CD11b (CC126 clone; Bio-Rad Laboratories Inc.) and an Accuri C6 flow cytometer (BD Biosciences).

The 50-mL tubes were centrifuged at 1,500 × *g* for 20 min at 4°C to collect somatic cells. Cells were resuspended in 50 mL of cold PBS and again centrifuged at 650 × *g* for 10 min at 4°C. Cell pellets were resuspended in 0.5 mL of Trizol Reagent (Invitrogen) and frozen at −80°C until subsequent RNA extraction. Total RNA was extracted using the Direct-zol TM-96 RNA Kit (Zymo Research). Total RNA (100 ng, 260/280 ratio = 1.8 ± 0.2) was reverse transcribed to cDNA using a high-capacity cDNA reverse transcription kit (Thermo Fisher Scientific). Quantitative PCR was performed using the CFX96 Real-Time System (Bio-Rad Laboratories Inc.) using conditions and primer sequences for *ACTINB*, *CCL5*, *DEFB7*, *GAPDH*, *IL1B*, *MT2A*, *NOS2A*, and *RPS9* as previously reported (Kweh et al., 2021). For determination of relative transcript abundance, the threshold cycle (**Ct**) for each gene in each cDNA sample was normalized to the geometric mean of *ACTB*, *GAPDH*, and *RPS9* Ct values using the equation ΔCt = Ct_(gene of interest)_ – Ct_(reference genes)_ based on the theory of Livak and Schmittgen (2001). The ΔCt values for each gene were used for statistical analysis and least squares means of ΔCt values were transformed by 2^−ΔCt^.

Data were analyzed with mixed models using the MIXED procedure of SAS (ver. 9.4, SAS Institute Inc.). For the main model, cow was used as the experimental unit and the model included fixed effects of treatment, time, and interaction between treatment and time. Cow within treatment was a random effect in the model. The Kenward-Roger method was used to calculate the correct denominator degrees of freedom to compute *F*-tests in the statistical models. Orthogonal contrasts were used to test effects of source (25D vs. 1,25D), dose (0 vs. 100 vs. 500 μg of 25D or 0 vs. 10 vs. 50 μg of 1,25D), and vitamin D treatment (CNTRL vs. all vitamin D treatments). Separate analyses also were performed to determine effects of vitamin D treatments on gene expression within cow. Each source and dose of vitamin D was analyzed as a separate experiment such that there were 4 experiments: CNTRL vs. 100 μg of 25D, CNTRL vs. 500 μg of 25D, CNTRL vs. 10 μg of 25D, and CNTRL vs. 50 μg of 1,25D, where CNTRL represents the placebo-treated quarters of each cow. In this case, the quarter within cow was used as the experimental unit and the models included treatment (vitamin D-treated quarters vs. placebo-treated quarters), time and interaction between treatment and time as fixed effects, and quarter within cow as random effect. Differences with *P* ≤ 0.05 were considered significant, and tendencies for differences were reported if 0.05 < *P* ≤ 0.10.

The 1,25D treatments increased (*P* < 0.05) concentrations of 1,25D and Ca in serum compared with CNTRL and 25D treatments (Figure 1). Serum 1,25D concentrations increased with the amount of 1,25D and were greatest immediately after treatment for cows receiving 50 μg of 1,25D (Figure 1A). Serum 1,25D concentrations of cows receiving 25D treatments did not differ from cows receiving CNTRL (Figure 1A). Serum Ca concentrations increased with amount of 1,25D treatment and were greatest at 24 h after treatment (Figure 1B). Serum Ca concentrations of cows receiving 50 μg of 1,25D treatments remained greater than CNTRL 48 h after treatments (Figure 1B). Serum Ca concentrations of cows receiving 25D treatments did not differ from CNTRL (Figure 1B). An interaction of treatment by time (*P* < 0.001) was observed for SCC in milk because cell counts of 1,25D-treated quarters increased by a greater increment at 6 h compared with CNTRL and 25D-treated quarters before decreasing at 12 and 24 h (Figure 1C). The percentages of neutrophils (34 ± 5%) and macrophages (22 ± 2) did not differ among treatments.

**Figure 1:**
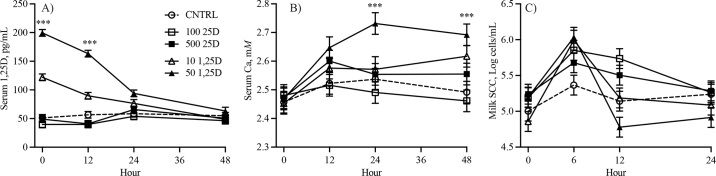
Serum concentrations of 1,25-dihydroxyvitamin D_3_ (1,25D, A) and Ca (B) and milk SCC (C) of dairy cows that received intramammary treatments of placebo control (CNTRL), 100 μg of 25-hydroxyvitamin D_3_ (100 25D), 500 μg of 25D (500 25D), 10 μg of 1,25D (10 1,25D), or 50 μg of 1,25D (50 1,25D) in 2 ipsilateral quarters (n = 4 cows/treatment). Data represent LSM ± SE. (A) Effect of treatment, *P* < 0.001; time, *P* < 0.001, and treatment by time, *P* < 0.001. ***Effect of treatment at indicated time, *P* < 0.001. Contrasts: linear effect of 25D, *P* = 0.26; linear effect of 1,25D, *P* < 0.001; 25D vs. 1,25D, *P* < 0.001. (B) Effect of treatment, *P* = 0.003; time, *P* < 0.001 and treatment by time, *P* = 0.08. ***Effect of treatment at indicated time, *P* < 0.001. Contrasts: linear effect of 25D, *P* = 0.40; linear effect of 1,25D, *P* = 0.002; 25D vs. 1,25D, *P* = 0.003. (C) Data represent quarter-level SCC for treated quarters. Effect of treatment, *P* = 0.01; time, *P* < 0.001; treatment by time, *P* < 0.001. Contrasts: linear effect of 25D, *P* = 0.005; linear effect of 1,25D, *P* = 0.51; 25D vs. 1,25D, *P* = 0.005.

Both sources of vitamin D increased (*P* < 0.05) transcripts of *CYP24A1*, *NOS2A*, and *CCL5* in milk somatic cells compared with CNTRL in a dose-dependent manner (Figure 2A–C). An interaction between treatment and time (*P* < 0.001) also was observed for *CYP24A1* because compared with CNTRL at 6 h, *CYP24A1* was increased (*P* < 0.01) by 6-, 73-, 237-, and 303-fold in 100 μg of 25D, 500 μg of 25D, 10 μg of 1,25D, and 50 μg of 1,25D-treated quarters, respectively, but at 24 h, the only treatment that differed from CNTRL was 500 μg of 25D (57-fold greater than control, *P* < 0.001). The 1,25D treatments and 500 μg of 25D treatment increased (*P* < 0.05) transcripts for *NOS2A* in milk somatic cells 3- to 4-fold compared with CNTRL (Figure 2B). The 25D and 1,25D treatments increased *CCL5* transcripts in milk somatic cells in similar linear fashions so that maximum transcript abundance was 3-fold greater compared with CNTRL (Figure 2C). Relative abundance for *DEFB7*, *IL1B*, and *MT2A* transcripts, which were previously reported to be upregulated by vitamin D signaling (Kweh et al., 2021) did not differ among treatments (Figure 2D-F).

**Figure 2:**
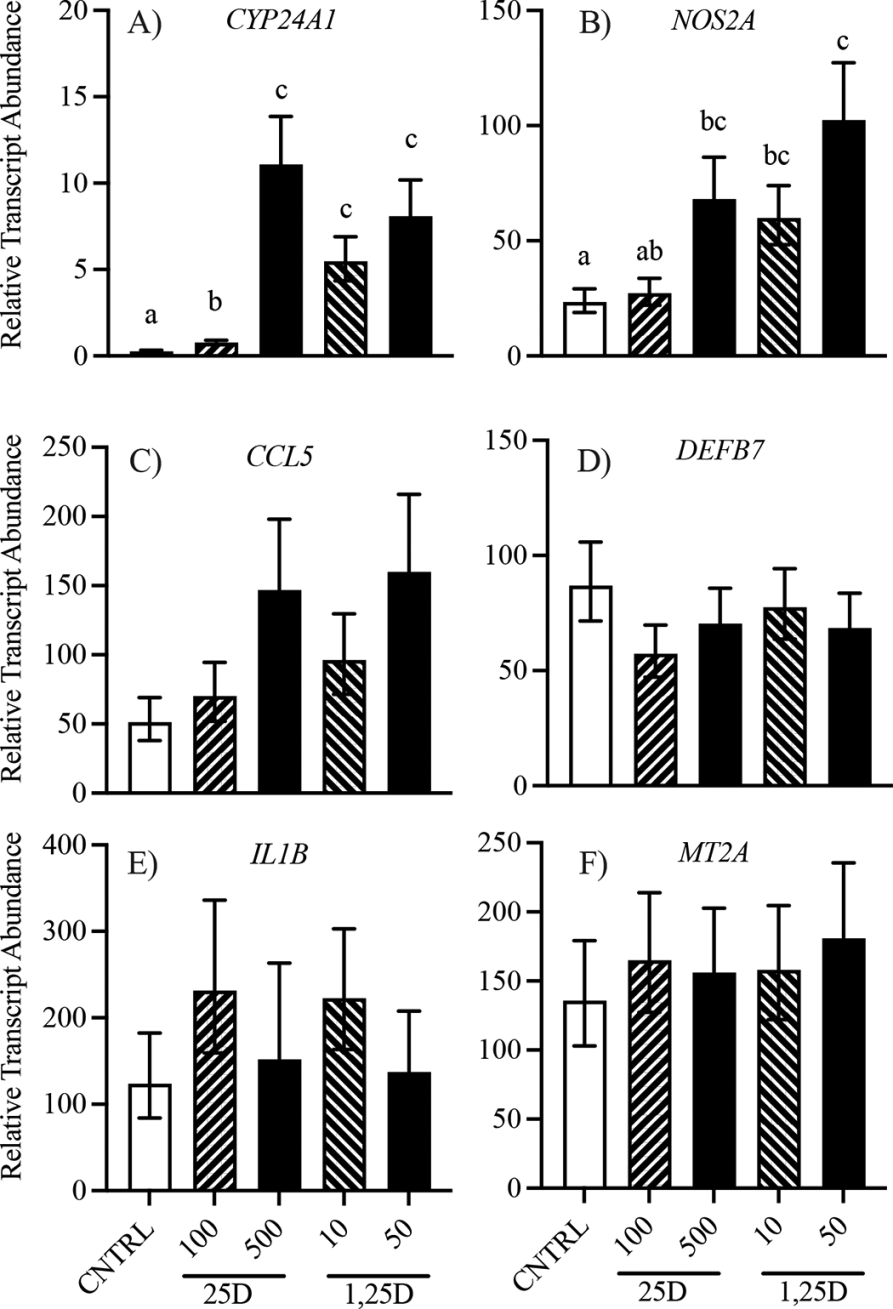
Expression of 24-hydroxylase (A, *CYP24A1*), inducible nitric oxide synthase (B, *NOS2A*), chemokine C-C motif ligand 5 (C, *CCL5*), β-defensin 7 (D, *DEFB7*), interleukin 1β (E, *IL1B*), and metallothionein (F, *MT2A*) gene in milk somatic cells of dairy cows that received intramammary treatments of placebo control (CNTRL), 100 μg of 25-hydroxyvitamin D_3_ (25D), 500 μg of 25D, 10 μg of 1,25-dihydroxyvitamin D_3_ (1,25D), or 50 μg of 1,25D in 2 ipsilateral quarters (n = 4 cows/treatment). Somatic cells were collected at 0, 6, 12, and 24 h relative to treatment. Data represent treatment LSM ± SE of transcript abundance relative to geometric mean of *ACTB*, *GAPDH*, and *RPS9* transcripts. (A) Effect of treatment, *P* < 0.001; means with different letters (a–c) are different, *P* < 0.05. Contrasts: linear effect of 25D, *P* < 0.001; linear effect of 1,25D, *P* < 0.001; 25D vs. 1,25D, *P* < 0.001. (B) Effect of treatment, *P* < 0.001; means with different letters (a–c) are different, *P* < 0.05. Contrasts: linear effect of 25D, *P* = 0.01; linear effect of 1,25D, *P* < 0.001; 25D vs. 1,25D, *P* = 0.01. (C) Effect of treatment, *P* = 0.05. Contrasts: linear effect of 25D, *P* = 0.04; linear effect of 1,25D, *P* = 0.01; 25D vs. 1,25D, *P* = 0.5. (D–F) Effect of treatment and contrasts, *P* > 0.1.

The vitamin D treatments increased *CYP24A1* and *NOS2A* transcripts in milk somatic cells of treated glands compared with placebo glands within treatment cows (Figure 3). For each amount and source of vitamin D, *CYP24A1* transcripts were increased (*P* < 0.001) by 6 h after treatment compared with placebo glands within cow (Figure 3A-D). Abundance of *CYP24A1* remained 27-fold greater (*P* < 0.001) than placebo glands at 24 h for cows receiving 500 μg of 25D (Figure 3B). In comparison, 50 μg of 1,25D induced a robust increase of *CYP24A1* at 6 h that dissipated by 24 h (Figure 3D).

**Figure 3:**
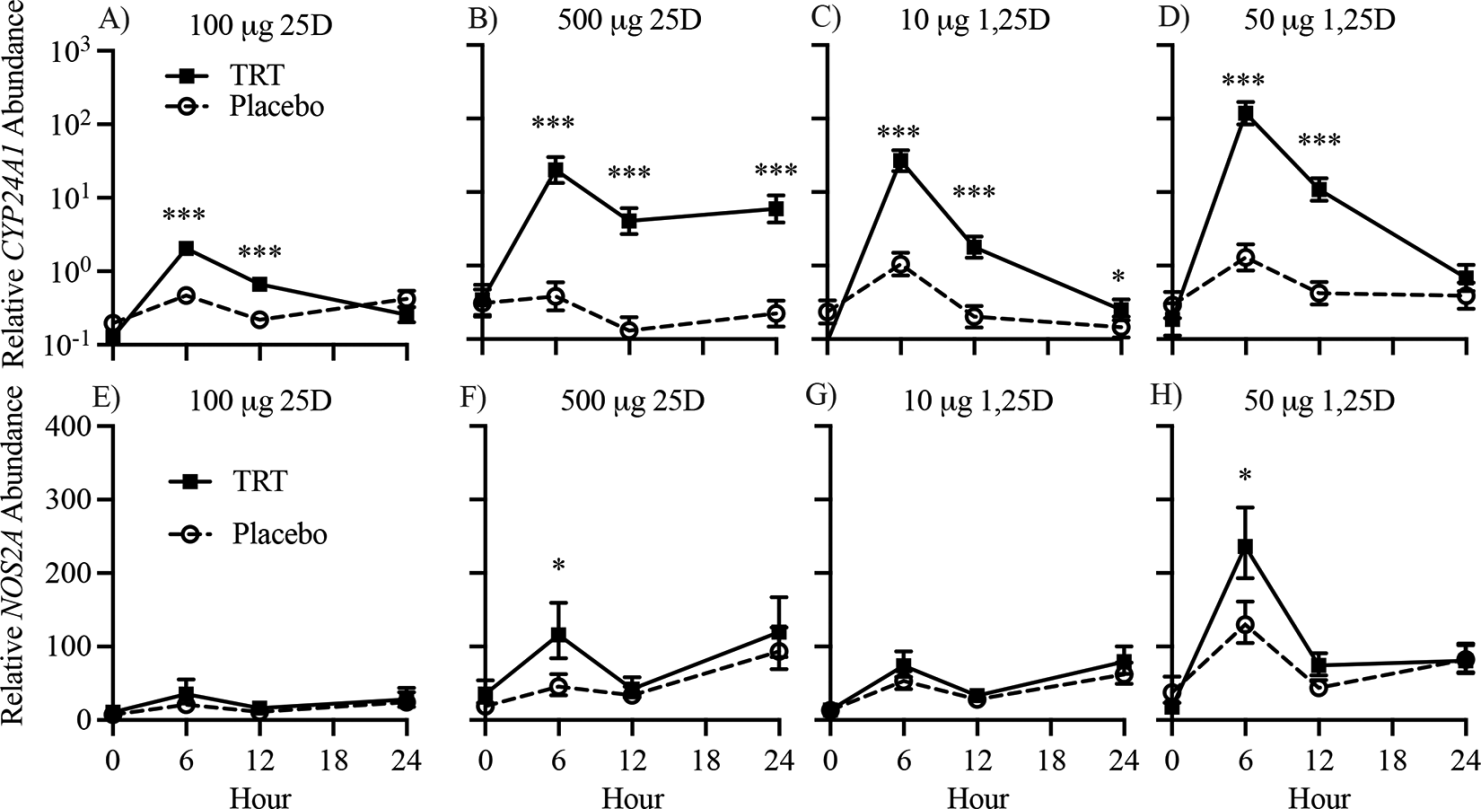
Expression of *CYP24A1* (A–D) and *NOS2A* (E–H) in milk somatic cells of mammary quarters treated (TRT) with 100 (A and E) or 500 (B and F) μg of 25-hydroxyvitamin D_3_ (25D) or 10 (C and G) or 50 (D and H) μg of 1,25-dihydroxyvitamin D_3_ compared with placebo control quarters within cow. The 25D and 1,25-dihydroxyvitamin D_3_ (1,25D) treatments were administered in in 2 ipsilateral quarters of 4 cows (n = 8 quarters/treatment), and the CNTRL treatments were administered in the opposite ipsilateral quarters (n = 8). Data represent treatment by time LSM ± SE of transcript abundance relative to the geometric mean of *ACTB*, *GAPDH*, and *RPS9* transcripts. *P < 0.05, ***P < 0.001: means differ at the indicated time.

The intracrine vitamin D pathway is activated in the mammary gland during mastitis and vitamin D signaling in immune and mammary epithelial cells of the mammary gland is believed to contribute to defense host-defense and protection of mammary tissues during IMI (Nelson et al., 2010a). Therefore, intramammary vitamin D treatments are a potential adjunctive therapy for mastitis in dairy cows. However, the major calcitropic effect of vitamin D (Hove et al., 1983) is a potential downside to intramammary vitamin D treatments.

Data from the present experiment show that intramammary 1,25D rapidly appeared in circulation and increased serum Ca concentrations. The 50 μg 1,25D treatments amounted to a total of 100 μg of 1,25D, which is approximately 30-fold more than the total amount of 1,25D in circulation of lactating cows in normal conditions (Horst and Littledike, 1982). The serum 1,25D concentrations of 1,25D-treated cows in this experiment were like those of postpartum dairy cows with hypocalcemia (Kichura et al., 1982). Subcutaneous injection of 100 μg of 1,25D resulted in a similar rapid increase in concentrations of serum 1,25D and Ca (Vieira-Neto et al., 2017). The 25D treatments, on the other hand, did not affect concentrations of 1,25D or Ca in serum. The 500 μg 25D treatments amounted to a total of 1 mg of 25D, which is approximately 25% of the total amount of 25D in circulation (Horst and Littledike, 1982). A small change in serum 25D from the 500 μg 25D treatments was possible, but elsewhere 100 μg intramammary 25D treatments did not affect serum 25D concentrations (Lippolis et al., 2011; Merriman et al., 2018). Moreover, large changes (i.e., 100 ng/mL of serum) did not affect concentrations of serum 1,25D, the main metabolite of concern (Poindexter et al., 2020). The data here show that intramammary 25D, but not 1,25D, can be administered without affecting concentrations of 1,25D and Ca in serum.

Intramammary 25D and 1,25D treatments elicited changes in gene expression in milk somatic cells like previously reported (Merriman et al., 2017, 2018). The 1,25D is a potent influencer of vitamin D-associated genes in immune cells (Wang et al., 2005). The *CYP24A1* is under control of multiple vitamin D response elements, so it serves as a prime indicator of vitamin D receptor activity (Meyer et al., 2010). Bovine *NOS2A*, *CCL5*, *DEFB7*, *IL1B*, and *MT2A* also are increased by 1,25D in bovine monocytes (Kweh et al., 2021). Here, 1,25D upregulated *CYP24A1*, *NOS2A*, and *CCL5* but not *DEFB7*, *IL1B*, and *MT2A*. Vitamin D activity is cell and context dependent (Pike and Meyer, 2014), so it is not surprising that *DEFB7*, *IL1B*, and *MT2A* were not upregulated in milk somatic cells. In contrast, 1,25D induced expression of *CYP24A1* and *NOS2A* in milk somatic cells, neutrophils, and mammary epithelial cells (Merriman et al., 2015). The effect of 1,25D on *CYP2A1* and *NOS2A* in 1,25D-treated glands was robust and dose dependent but short lived. Metabolism of 1,25D to 24-hydroxyvitamin D metabolites by CYP24A1 and rapid diffusion of 1,25D into circulation provide a reasonable explanation for the pattern of *CYP24A1* and *NOS2A* that was observed. Some of the changes in gene expression could be due to difference in presence of somatic cells in the mammary gland after treatment. However, expression of *CYP24A1* in blood leukocytes is very low (Merriman et al., 2018), so it is unlikely that the increase in *CYP24A1* at 6 h resulted from an influx of leukocytes in 1,25D-treated quarters. On the other hand, the 1,25D treatments also appeared to increase *CYP24A1* in placebo-treated glands, which is in line with the systemic effects observed for serum Ca concentrations in 1,25D-treated cows.

Like 1,25D treatments, the 25D treatments upregulated milk cell *CYP24A1*, *CCL5*, and *NOS2A* in a dose-dependent manner. Merriman et al. (2018) reported that 100 μg intramammary 25D treatment increased milk cell *CYP24A1* and *NOS2A*. In an experiment with dietary 25D, *CYP24A1*, *DEFB7*, *IL1B*, and *NOS2A* transcripts in milk samples of healthy glands were positively correlated with serum 25D concentrations in lactating dairy cows (Poindexter et al., 2020). Immune cell 1α-hydroxylase activity converts 25D to 1,25D, thereby providing 1,25D for intracrine and paracrine signaling without affecting vitamin D endocrine signaling (Adams and Hewison, 2008). For example, treatment of monocytes with 0 to 100 ng/mL 25D resulted in a linear increase of *CCL5*, *DEFB7*, and *NOS2A* expression (Nelson et al., 2010b). The 25D treatments did not increase *NOS2A* expression to the same extent as 1,25D even though the effect of 500 μg of 25D on *CYP24A1* was similar to 1,25D treatments. In addition, the effect of 500 μg of 25D on *CYP24A1* seemed to be more prolonged compared with 1,25D treatments.

The effect of 25D on host-defense genes like *NOS2A* would probably be more pronounced in activated immune cells during mastitis due to more 1α-hydroxylase activity and synergy between vitamin D and toll-like receptor signaling (Liu et al., 2007). Cytokine and toll-like receptor signaling upregulate *CYP27B1* and subsequent 1α-hydroxylase activity in monocytes (Stoffels et al., 2006). Intramammary *Streptococcus uberis* infection and LPS challenge upregulated *CYP27B1* expression in milk macrophages and neutrophils of dairy cows (Nelson et al., 2010a; Merriman et al., 2018). Conversely, toll-like receptor and interferon-γ signaling suppress *CYP24A1* expression and subsequent inactivation by 24-hydroxylases (Vidal et al., 2002; Kweh et al., 2021). Convergence of toll-like receptor and vitamin D signaling pathways also potentiated nitric oxide production and antimicrobial activity of macrophages (García-Barragán et al., 2018).

Strengthening vitamin D signaling of the mammary gland with 25D treatments seems to be a promising strategy to improve defense of the mammary gland against pathogens and tissue damage associated with mastitis. Overall, vitamin D signaling in immune cells increases antimicrobial and antioxidant activity while acting to contain inflammatory responses (Chen et al., 2015; García-Barragán et al., 2018; Dai et al., 2019). Notably, dietary and intramammary 25D treatments reduced symptoms of mastitis severity in dairy cows (Lippolis et al., 2011; Poindexter et al., 2020). Collectively, the data show that intramammary 25D potentiated vitamin D signaling in milk cells, but unlike intramammary 1,25D, did not affect serum Ca. Therefore, intramammary 25D seems to be a suitable adjunctive treatment for mastitis in dairy cows.
